# Shaping Microstructure and Mechanical Properties of High-Carbon Bainitic Steel in Hot-Rolling and Long-Term Low-Temperature Annealing

**DOI:** 10.3390/ma14020384

**Published:** 2021-01-14

**Authors:** Tomasz Dembiczak, Marcin Knapiński

**Affiliations:** 1 Faculty of Science and Technology, Jan Dlugosz University in Czestochowa, 13/15 Armii Krajowej Street, 42-200 Czestochowa, Poland; 2 Faculty of Production Engineering and Materials Technology, Czestochowa University of Technology, 19 Armii Krajowej Street, 42-201 Czestochowa, Poland; marcin.knapinski@pcz.pl

**Keywords:** high-carbon bainitic steel, dynamic and post-dynamic softening, hot-rolling, final microstructure and mechanical properties

## Abstract

Based on the research results, coefficients in constitutive equations, describing the kinetics of dynamic, meta-dynamic, and static recrystallization in high-carbon bainitic steel during hot deformation were determined. The developed mathematical model takes into account the dependence of the changing kinetics in the structural size of the preliminary austenite grains, the value of strain, strain rate, temperature, and time. Physical simulations were carried out on rectangular specimens. Compression tests with a flat state of deformation were carried out using a Gleeble 3800. Based on dilatometric studies, coefficients were determined in constitutive equations, describing the grain growth of the austenite of high-carbon bainite steel under isothermal annealing conditions. The aim of the research was to verify the developed mathematical models in semi-industrial conditions during the hot-rolling process of high-carbon bainite steel. Analysis of the semi-industrial studies of the hot-rolling and long-term annealing process confirmed the correctness of the predicted mathematical models describing the microstructure evolution.

## 1. Introduction

The dynamic development of modern technologies of the plastic processing of metals is aimed at, in addition to giving the products the required shape, obtaining the most favorable material structure that has a significant impact on the mechanical properties of the product. Knowledge of the phenomena occurring during the plastic deformation of metal at high temperature, both in the deformation gap and outside it, allows the structural changes taking place in the processed material to be controlled. The modeling of thermo-plastic processing processes is currently one of the most effective research methods used in the optimization of the technological parameters of these processes. The most important advantage of this method is that a quantitative description of the physical phenomena is obtained, the course of which, during plastic processing, shapes the final microstructure and mechanical properties of steel products. It also enables analysis of the influence of the evolution of austenite grains in the metal forming phase at high temperature on the dynamics of their decomposition during cooling and the obtained microstructure of the product after cooling to ambient temperature. Currently, the development and application of new materials and technologies requires a rapid transition from calculating the theoretical results and research on a laboratory scale to industrial implementation. Industrial research is the last, but usually very expensive, element of the implementation process. These costs can be reduced substantially, and the process itself can be significantly simplified and accelerated using modern methods of mathematical and physical modeling. There are many works on mathematical and physical modeling available in the scientific literature, examples may be works [1,2,3,4,5,6,7,8,9,10].

The main objective of the research, the results of which are presented in the paper, was to comprehensively develop numerical models of dynamic, meta-dynamic, and static recrystallization phenomena occurring in alloy austenite during hot plastic working. High-carbon bainitic steel, for which the final mechanical properties are obtained by long-term low-temperature isothermal annealing, was tested. The product shaping process takes place at high temperatures, most often by rolling flat products. For the correct design of such a process, and in particular to determine the morphology of austenite after rolling, an important element is the knowledge of the kinetics of the recovery processes of the hot-deformed microstructure. The paper presents proprietary models of dynamic, meta-dynamic, and static recrystallization kinetics of alloy austenite under the conditions of hot plastic working. Changes in austenite grain size related to recrystallization processes as well as the kinetics of its isothermal growth were also determined. The numerical models developed on a laboratory scale using the Gleeble system were verified in rolling conditions with the use of a semi-industrial technological line for sheet rolling (LPS). The presented original research results are an added value to the current state of knowledge in publications on the numerical models of the kinetics of the alloy austenite recrystallization processes. The given numerical values of the equation coefficients enable other scientists or engineers to model the recrystallization processes of the tested steel. They supplement the database, which has been created for many years, on the quantitative assessment of recrystallization phenomena occurring during hot plastic working.

The final mechanical properties of high-carbon bainitic steel are obtained by long-term low-temperature isothermal annealing. During this process, carbide-free bainite is formed in the microstructure in the form of strips with a thickness of 20–100 nanometers, which, together with the residual austenite showing the TRIP effect, ensures very high strength with good plasticity of the product. Work on the understanding of the phenomena related to obtaining particularly high mechanical properties in steels of this type developed in the first decade of the 21st century [11,12,13,14], but the available literature does not contain much information on this subject, probably due to the potential use of this material for military purposes. The paper also presents the results of tests of selected mechanical properties of heavy plates from the analyzed steel rolled in various conditions after low-temperature annealing for 70 h.

## 2. Materials and Methods

### 2.1. Material Descriptions

The material used for the study was high-carbon bainitic steel developed in the Łukasiewicz Research Network—Institute of Iron Metallurgy in Gliwice [11], using the results of basic research on bainitic transformation and bainite structure [12,13,14,15,16,17]. The new generation bainitic steel is an alloy steel containing, depending on the intended use, 0.5 to 0.85% C, additions of Si, Mn, Co, Mo, Cr, and micro-additives of other elements. The use of this composition allows very high hardness in the range of 600–700 HV and high strength up to 2.5 GPa to be obtained, while maintaining good plastic properties. The special feature of this steel is its high impact resistance, which is why its main application may be ballistic shields. The use of ballistic shields made of high-carbon bainitic steel makes it possible to reduce the weight of the shields, leading to material and energy savings. Newly developed steel, in addition to applications in the defense industry, can also be used as a construction material, impact and abrasion resistant layers in coal and rock mining, as well as protection against explosion and rock impact. High-carbon bainitic steel is a new material, not yet produced in Poland on an industrial scale. In Great Britain, industrial trials with steels in this class have been successfully completed. The introduction of a new generation bainitic steel for industrial production in Poland is planned in the coming years. Table 1 presents the chemical composition of high-carbon bainitic steel (Łukasiewicz Research Network—Institute of Iron Metallurgy in Gliwice), which was the basic research material.

### 2.2. Plastic Flow Curves

Plastic flow curves for the studied steel were determined for the following temperatures: 900, 950, 1000, 1050 and 1100 °C, strain rate: 0.1; 1 and 10 s^−1^ and true strain 0–1. To determine the plastic flow curves of the steel, plastometric tests were carried out using a Gleeble 3800 physical metallurgical processes simulator with a Hydrawedge II module. For the plastometric tests, rectangular samples with dimensions in millimeters of 10 × 15 × 20 were used. The plastic flow curves were determined in compression tests with a flat deformation state, reflecting the conditions of the hot-rolling process.

### 2.3. Methodology for Developing Constitutive Equations Describing Kinetics of Dynamic Recrystallization

The phenomenon of dynamic recrystallization occurring in the microstructure during the hot deformation of steel was analyzed in two stages in the temperature range of 900–1100 °C and at the strain rate of 0.1–10 s^−1^. In the first stage, based on the developed plastic flow curves, considering the temperature correction and strain rate, the deformation parameters necessary to initiate the phenomenon of dynamic recrystallization were determined. The dependence of the coefficient strengthening intensity θ as a function of stress was determined, describing the behavior of the material during hot plastic deformation [18].

In the second stage of the mathematical modeling of dynamic recrystallization phenomena, the coefficients found in constitutive equations ε_p_, ε_kr_, ε_0.5_ and d_DRX_ were determined, considering the influence of the temperature, strain rate described by the Zener–Hollomon (Z) parameter and the initial austenite grain size.

### 2.4. Methodology for Developing Constitutive Equations Describing Kinetics of Meta-Dynamic Recrystallization

The kinetics of meta-dynamic recrystallization were analyzed using the stress relaxation method. This method involves performing a single plastic deformation, after which the sample is held between anvils with the real-time recording of the decrease in force. After plastic deformation, during which dynamic recrystallization was initiated, the material undergoes a process of meta-dynamic recrystallization, as a result of which the value of internal stress decreases. The advantage of this method is that the full course of the recrystallization kinetics curve is obtained with only one compression test for selected plastic deformation conditions.

Experimental studies aimed at developing the constitutive equations of meta-dynamic recrystallization were carried out for a flat state of strain, using rectangular samples with dimensions in millimeters of 10 × 15 × 20, at temperatures of 900 °C, 1000 °C, 1100 °C, with a given plastic strain determined based on the model of dynamic recrystallization kinetics, and at strain rates of 0.1 s^−1^ and 10 s^−1^. After deformation, the samples remained under load for 10 s, while recording a decrease in pressure force reflecting stress relaxation in high-carbon bainitic steel. The tests were carried out using the Gleeble 3800 physical simulator. The experimental research was divided into two stages.

In the second stage of mathematical modeling research, the coefficients were determined in the constitutive equation describing the time of meta-dynamic half-recrystallization t_0.5MDRX_ as a function of the strain rate, temperature, and activation energy. The values of the coefficients appearing in the constitutive equation describing the time t_0.5MDRX_ and the material constant were determined by logarithmizing both sides of the equation. Then, depending on the relationship between ln t_0.5MDRX_ and ln $\dot{\varepsilon }$ and ln t_0.5MDRX_ and ln 1/T, the ascending coefficients were determined in the constitutive equation.

Based on metallographic tests, a constitutive equation of the mathematical model describing the size of meta-dynamically recrystallized grains d_MDRX_ was developed as a function of the Zener–Hollomon parameter. The coefficients appearing in this equation were determined from the relation between ln d_MDRX_ and ln Z.

### 2.5. Methodology for Developing Constitutive Equations Describing Kinetics of Static Recrystallization

The kinetics analysis of static recrystallization was carried out using the stress relaxation method. The experimental tests were performed for the temperatures of 950 °C and 1050 °C, with given strains of 0.05 and 0.10, and at strain rates of 0.1 s^−1^ and 10 s ^−1^. After deformation, the samples remained under load for 100 s. The tests were carried out using the Gleeble 3800 device and divided into two stages.

In the first stage, the analysis of the kinetics of static crystallization based on the Avrami equation makes it possible to determine the time t_0,5SRX_ needed to recrystallize 50% of the material volume.

In the second stage of the tests coefficients were determined in the constitutive equation describing the time of half-static recrystallization t_0.5SRX_ as a function of: strain, strain rate, initial austenite grain size, and activation energy. The values of the coefficients appearing in the constitutive equation describing time t_0.5SRX_ and the material constant were determined after logarithmizing both sides of the equation. Then, depending on the relationships: ln t_0.5SRX_ from ln ε, ln t_0.5SRX_ from ln $\dot{\varepsilon }$, ln t_0.5SRX_ from ln d_0_ and ln t_0.5SRX_ from ln 1/T, ascending coefficients were determined in the constitutive equation. Exponent n_3_ appearing in the equation describing the kinetics of static recrystallization was determined from the relation between ln (ln (1/1 − X)) and ln t.

Based on metallographic examination, the constitutive equation of the model describing the size of statically recrystallized grains d_SRX_ as a function of the initial austenite grain size, strain, strain rate, and activation energy was developed. The coefficients appearing in this equation were determined from the relation: ln d_SRX_ from ln d_0_, ln d_SRX_ from ln ε, ln d_SRX_ from ln $\dot{\varepsilon }$, ln d_SRX_ from ln 1/T.

### 2.6. Methodology for Developing Constitutive Equations Describing Growth of Alloy Austenite Grains

To develop a constitutive equation describing the growth of the alloy austenite grains, tests were conducted under isothermal heating conditions. The tests consisted of heating the samples to a given temperature, holding them for a specified period of time and rapid cooling leading to freezing of the structure. Then, metallographic tests were performed to determine the size of the austenite grains. The tests were carried out for temperatures of 900 °C, 1000 °C and 1100 °C. The samples were held for 0, 10, 30, 60, 90 min. The tests were carried out on cylindrical samples with dimensions in millimeters ϕ 5 × 10 using a DIL 805 A/D dilatometer. Based on the conducted tests, constitutive equations describing the grain growth of high-carbon bainitic steel under isothermal heating conditions were developed.

### 2.7. The Process of Rolling High-Carbon Bainitic Steel in Industrial Conditions

The aim of the research on a semi-industrial scale was to verify the mathematical models describing the evolution of the microstructure during the hot-rolling process of high-carbon bainite steel. The tests were carried out for three variants with the end temperatures of rolling: 950, 900 and 850 °C. After rolling, the end section of the strand, approximately 40 cm long, was quenched in a bath filled with water to freeze the structure. The strand at 250 °C was placed in an oven heated to 250 °C and annealed for 70 h for low-temperature heat treatment.

## 3. Results

Table 2 shows the results of the metallographic examinations under which the primary austenite grain size was defined.

During analysis of the plastic flow curves, a problem arises from the uncontrolled temperature increase of the sample resulting from strain occurring especially at strain rates higher than 5 s^−1^. The mathematical description of the plastic flow curves using Equation (1) makes it possible to introduce a temperature correction [19,20,21]:

(1) $$\sigma_{p}=f\left(\varepsilon ,\;\dot{\varepsilon }\right)e^{\frac{Q}{\mathrm{RnT}}}$$

where: ε—stres, $\dot{\varepsilon }$—strain rate Q—activation energy, R—gas constant, T—temperature, n—material constant.

**Figure 1:**
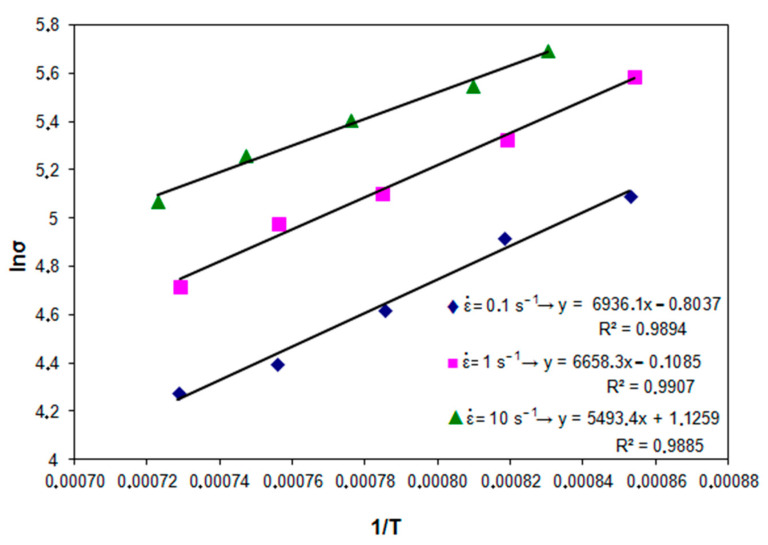
Relationship between yield stress and temperature for different strain rates.

By logarithmizing Equation (1) for the constant strain value and strain rate 0.1; 1 and 10 s^−1^, a relationship was obtained between the logarithm of the metal flow stress and the reciprocal of the temperature. Based on the performed linear regression for the relationship ln (σ) − 1/T (Figure 1), the activation energy was determined for high-carbon bainitic steel.

**Figure 2:**
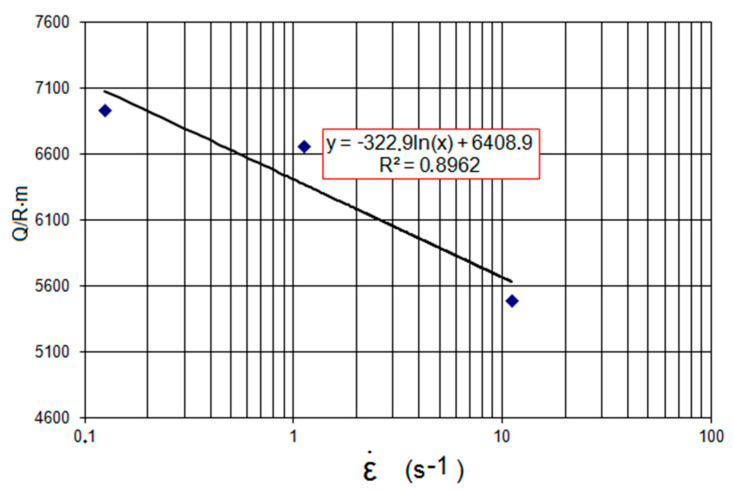
Change in activation energy as a function of strain rate from linear regression equation.

Figure 2 shows the relationship between the activation energy and the strain rate. It was assumed that for the researched steel the dependence of the activation energy on the strain rate is logarithmic [22,23].

Finally, the following equation was used to describe the influence of the temperature and strain rate:

(2) $$\sigma_{p}=f\left(\varepsilon ,\;\dot{\varepsilon }\right){\dot{\varepsilon }}^{\frac{c_{1}}{T}}e^{\frac{c_{2}}{T}}$$

where: *c*_1_, *c*_2_—coefficients dependent on the material, experimentally determined

**Figure 3:**
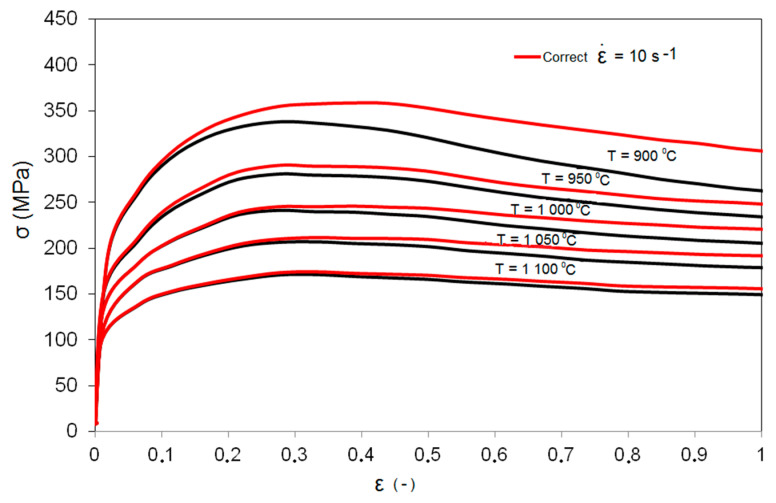
Plastic flow curves of high-carbon bainitic steel at strain rate of 10 s^−1^ at a temperature of 900–1100 °C.

The values of the coefficients (*c*_1_ = −322.89, *c*_2_ = 6408.9) and function f were determined from the regression equations and the measurement data provided in Figure 2. The coefficients developed in Equation (2) made it possible to correct the metal flow stress for the given deformation value, assumed temperature, and strain rate. An example of the course of an adjusted plastic flow curve of high-carbon bainitic steel is shown in Figure 3.

By analyzing the obtained plastic flow curves, it can be concluded that in the examined temperature range and strain rate 10 s^−1^, dynamic recrystallization in the steel takes place after exceeding the critical deformation value, and following this, the curves fall slightly in the directions of lower stress values, going into a steady state. This phenomenon is the dominant factor in the reconstruction of the structure.

### 3.1. Dynamic Recrystallization

The developed material coefficients in the mathematical models describing the phenomenon of dynamic recrystallization in high-carbon bainite steel are presented in detail in the authors’ research [24].

The determined coefficients in the constitutive equation describing peak strain ε_p_ for the investigated steel are as follows [18,24,25,26,27,28]:

(3) $$\varepsilon_{p}=0.0656d_{0}^{-0.2338}Z^{0.0634}$$

Critical strain ε_kr_ and peak strain ε_p_ are related by the material constant c, the experimental method of determination of which is shown in the work [24], and the mutual dependence of these strains is described by the following equation:

(4) $$c=\frac{\varepsilon_{\mathrm{kr}}}{\varepsilon_{p}}=0.6220$$

The value of constant *c* is an average value quotient of ε_kr_/ε_p_, determined for temperatures of 900–1100 °C and strain rates 0.1, 1 and 10 s^−1^. The constitutive equation defining critical strain ε_kr_ was determined based on Equations (3) and (4) and finally has the following forms [24]:

(5) $$\varepsilon_{\mathrm{kr}}=0.0408d_{0}^{-0.2338}Z^{0.0634}$$

Finally, for the investigated steel, strain ε_0.5_ was described by the equation [24]:

(6) $$\varepsilon_{0.5}=0.1333d_{0}^{-0.2434}Z^{0.064}$$

The kinetics of dynamic recrystallization is described as follows [24]:

(7) $$X_{\mathrm{DRX}}\left(t\right)=1-\exp\left[-4.3802{\left(\frac{\varepsilon -\varepsilon_{\mathrm{kr}}}{\varepsilon_{0.5}-\varepsilon_{\mathrm{kr}}}\right)}^{2.2394}\right]$$

The equation describing the average size of dynamically recrystallized grains is described in the paper [24] as follows:

(8) $$d_{{}_{\mathrm{DRX}}}=11111.1Z^{-0.1535}$$

### 3.2. Meta-Dynamic Recrystallization

The value of plastic deformation in the experimental tests was selected based on the developed model describing the kinetics of dynamic recrystallization (Equation (7)) for two strain rates 0.1 and 10 s^−1^. The development of a model describing the kinetics of this recrystallization in high-carbon bainite steel was carried out in two stages.

**Figure 4:**
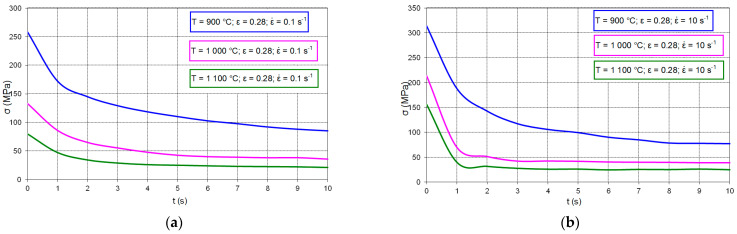
Stress relaxation of high-carbon bainitic steel in temperature range 900–1100 °C and plastic deformation 0.28: (**a**) for strain rate 0.1 s^−1^, (**b**) strain rate 10 s^−1^.

In the first stage, the change in the steel residual stresses as a function of time from the end of plastic deformation was analyzed using the stress relaxation method. Figure 4 shows examples of the curves of the steel residual stresses changes occurring during tests at temperatures of 900, 1000 and 1100 °C after plastic deformation of 0.28 set at the strain rates of 0.1 and 10 s^−1^.

To evaluate the phase meta-dynamically recrystallized after time (t), the softening coefficient was used, defined as follows:

(9) $$X_{m_{\mathrm{MDRX}}}\left(t\right)=\left(\frac{\sigma_{\mathrm{rs}}-\sigma_{\mathrm{rt}}(t)}{\sigma_{\mathrm{rs}}-\sigma_{\mathrm{rf}}}\right)$$

where: σ_rs_—initial stress value on the stress relaxation curve, σ_rt_ (t)—stress value during time relaxation (t), σ_rf_—final stress value after completion of the relaxation process.

Table 3 shows examples of the calculated values of the softening coefficient of the investigated steel.

Based on the softening fraction, the kinetics of meta-dynamic recrystallization were developed according to the Avrami equation:

(10) $$X=1-e^{\left(-kt^{n}\right)}$$

where: X—fraction of the recrystallized phase, t—time (s), k, n—coefficients for a given material grade determined experimentally

**Figure 5:**
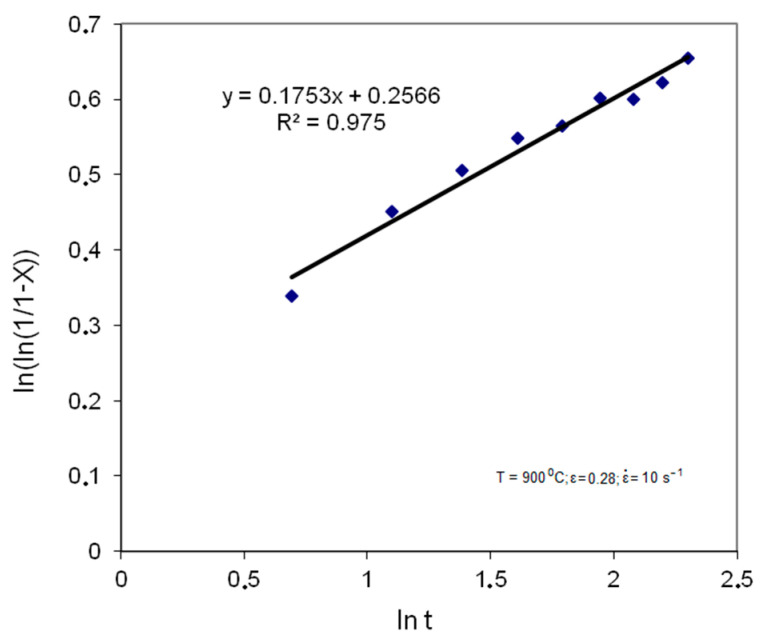
Dependence of ln (ln (1/1 − X)) on ln t for T = 900 °C; ε = 0.28; $\dot{\varepsilon }$ = 0.1 s^−1^.

Figure 5 shows the dependencies based on which coefficients (k) and (n) were determined for the temperature of 900 °C, strain 0.28 and strain rate 0.1 s^−1^.

The determined coefficients in the Avrami equation made it possible to describe the course of meta-dynamic recrystallization of the examined steel as a function of time for the deformation temperatures 900, 1000, 1100 °C and strain rates 0.1 and 10 s^−1^. Based on the quantitative description of the kinetics of meta-dynamic recrystallization, the time needed for 50% of the material to recrystallize was determined. In the next stage of the research, the determined times of half-recrystallization were used to develop a constitutive equation describing t_0.5MDRX_ as a function of the strain rate and activation energy. Table 4 shows the determined Avrami equation coefficients for the researched steel at temperatures 900, 1000, 1100 °C, strain 0.28 and strain rate 0.1 s^−1^.

**Figure 6:**
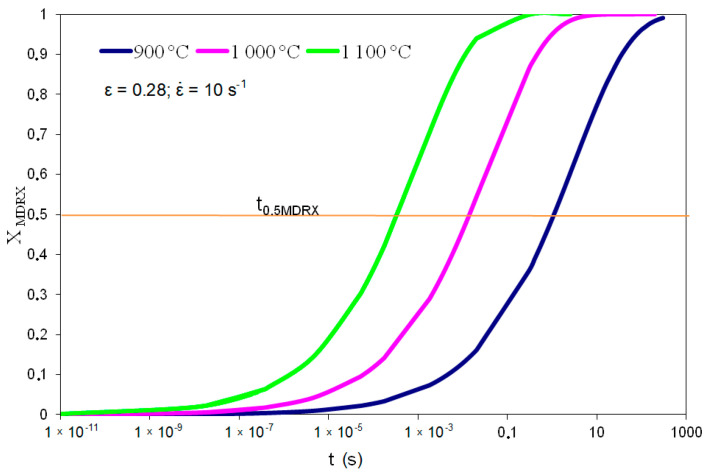
Kinetics of meta-dynamic recrystallization in high-carbon bainite steel based on Avrami equation.

Figure 6 shows examples of meta-dynamic recrystallization determined based on the Avrami equation for T = 900, 1000, 1100 °C and $\dot{\varepsilon }$ = 0.1 s^−1^.

**Figure 7:**
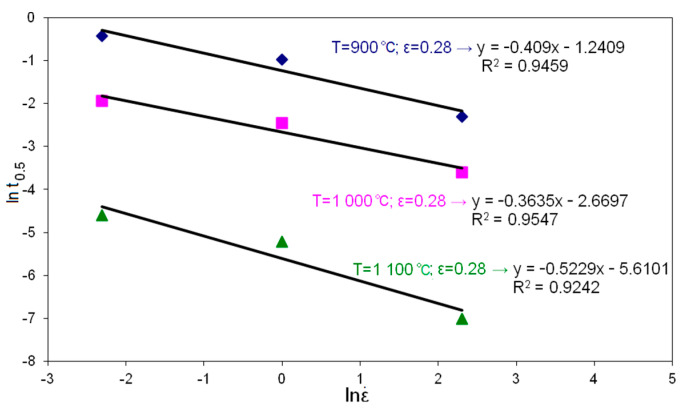
Dependence of half time of meta-dynamic recrystallization (ln t_0.5_) on strain rate (ln $\dot{\varepsilon }$).

**Figure 8:**
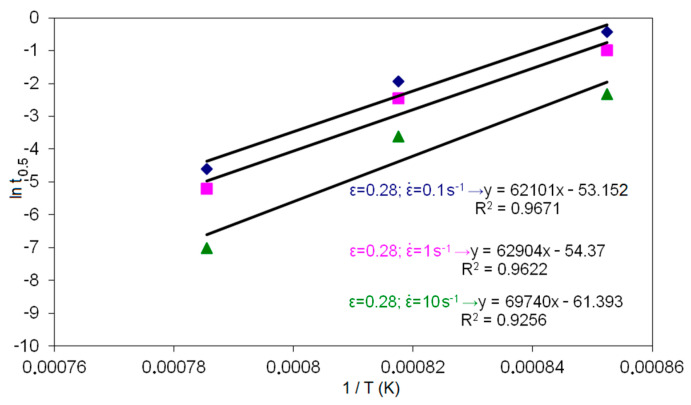
Dependence of half time of meta-dynamic recrystallization (ln t_0.5_) on temperature (1/T).

In the second stage of the research, the material constants appearing in the constitutive equation describing the time of half meta-dynamic recrystallization t_0.5MDRX_ in high-carbon bainite steel were determined. Figure 7 and Figure 8 show the dependencies used to determine the coefficients in t_0.5MDRX_.

The time needed to obtain 50% of the meta-dynamically recrystallized phase in high-carbon bainite steel is described as follows:

(11) $$t_{0.5_{\mathrm{MDRX}}}=5.05\times 10^{-24}{\overset{}{\dot{\varepsilon }}}^{-0.4318}e^{\left(\frac{539703.3}{\mathrm{RT}}\right)}$$

**Figure 9:**
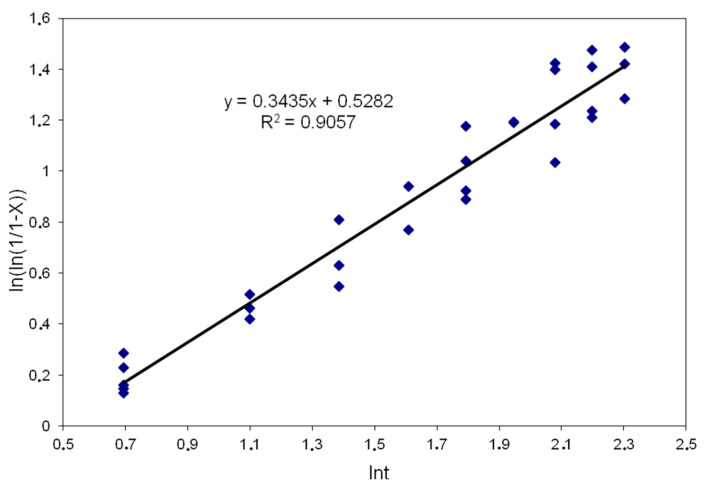
Relationship ln (ln (1/1 − X)) to ln t in range T = 900–1100 °C; ε = 0.28; $\dot{\varepsilon }$ = 0.1–10 s^−1^.

The exponent in the equation describing the kinetics of meta-dynamic recrystallization was determined based on the dependence presented in Figure 9.

The conducted research made it possible to determine all the coefficients of Equation (12) describing the amount of meta-dynamically recrystallized phase of material X_MDRX_ depending on the elapsed time (t) and considering the time t_0.5MDRX_ needed to obtain 50% of the meta-dynamically recrystallized phase in material.

(12) $$X_{\mathrm{MDRX}}=1-\exp\left[-0.6931\cdot {\left(\frac{t}{t_{0.5_{\mathrm{MDRX}}}}\right)}^{0.3435}\right]$$

**Figure 10:**
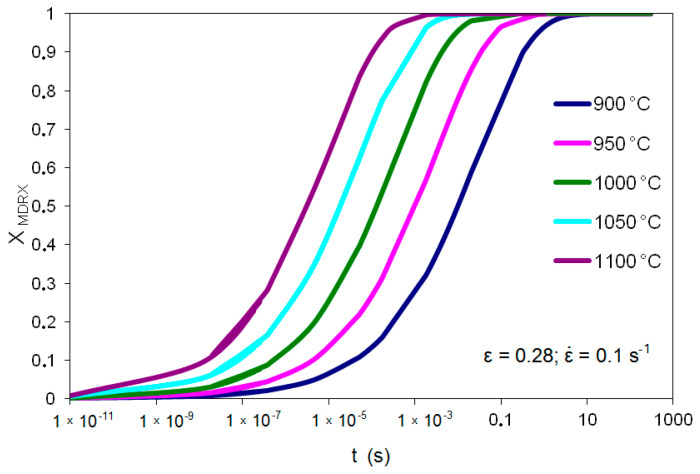
Kinetics of meta-dynamic recrystallization of high-carbon bainitic steel for T = 900–1100 °C, $\dot{\varepsilon }$ = 0.1 s^−1^.

Figure 10 shows the kinetics of meta-dynamic recrystallization for high-carbon bainite steel according to the models described in Equations (11) and (12).

**Figure 11:**
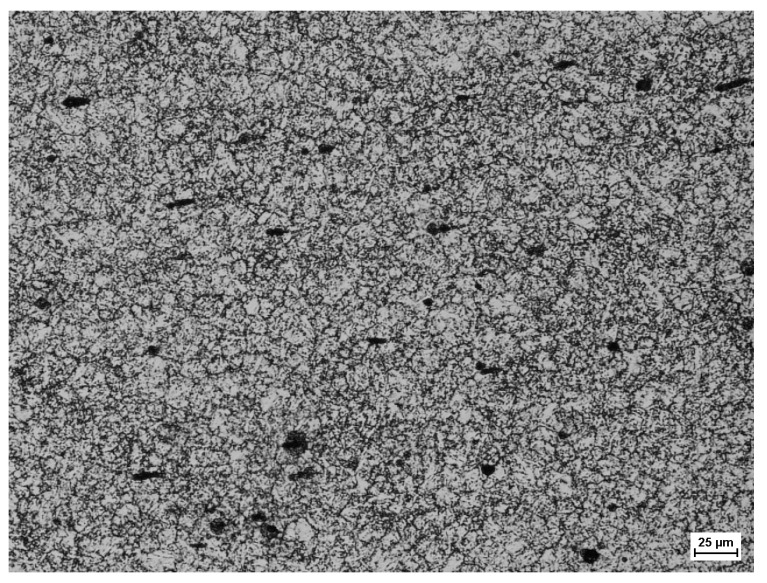
Microstructure of sample after experimental tests for T = 900 °C; ε = 0.28; $\dot{\varepsilon }$ = 0.1 s^−1^ dγ = 23.7 μm; area 200×; etched with picric acid (C_6_H_3_N_3_O_7_).

To determine the material coefficients of the model describing the grain size of meta-dynamically recrystallized austenite, metallographic tests were carried out after the performed experimental tests. Figure 11 shows an example of the microstructure of the meta-dynamically recrystallized alloy austenite grains obtained from the experimental tests carried out. 1. 

**Figure 12:**
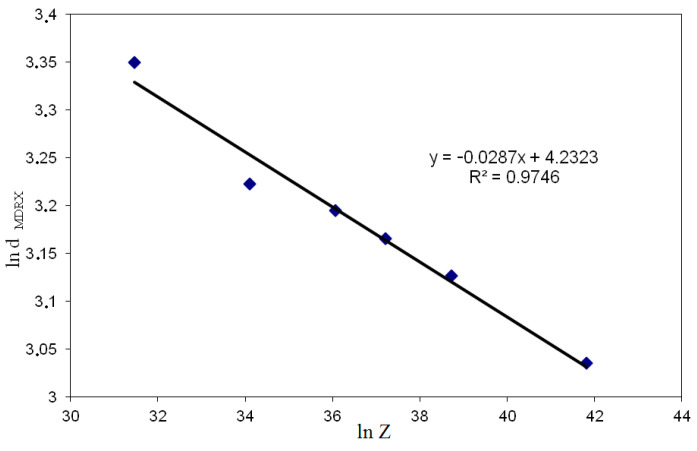
Dependence of meta-dynamically recrystallized austenite grains (ln d_MDRX_) on Zener–Hollomon parameter (ln Z).

Figure 12 shows the dependence based on which the material constants were determined in the model describing the size of the meta-dynamically recrystallized grains in high-carbon bainite steel.

The model describing the size of the meta-dynamically crystallized grains for the studies steel is as follows:

(13) $$d_{\mathrm{MDRX}}=68.875Z^{-0.0287}\;(\mu m)$$

### 3.3. Static Recrystallization

The value of plastic deformation in the experimental tests was selected based on analysis of the developed model describing the kinetics of the phenomenon of dynamic recrystallization and critical deformation described in the authors’ work [24]. Based on these data, it was found that only static recrystallization occurs in the material if it is deformed to a plastic deformation value not greater than 0.1. The development of a model describing the kinetics of static recrystallization in high-carbon bainite steel was carried out in two stages.

**Figure 13:**
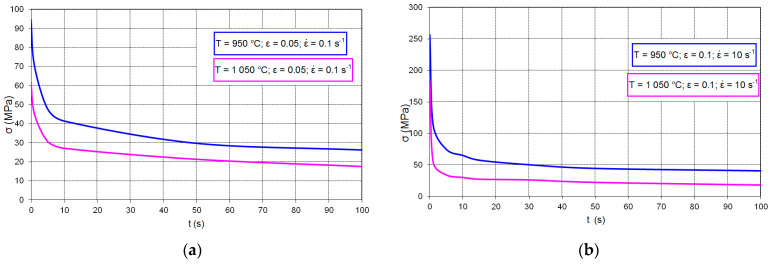
Stress relaxation of high-carbon bainite steel for temperature 950 and 1050 °C: (**a**) ε = 0.05; $\dot{\varepsilon }$ = 0.1 s^−1^, (**b**) ε = 0.15; $\dot{\varepsilon }$ = 10 s^−1^.

In the first stage of the research, an analysis of the change in residual stress as a function of time from the moment of the end of plastic deformation was carried out using the stress relaxation method. Figure 13 show examples of the curves of the steel residual stress changes occurring during the tests at the temperatures of 950 and 1050 °C after plastic deformation of 0.05 and 0.1 at the strain rates of 0.1 and 10 s^−1^.

To evaluate the amount of statically recrystallized phase after time (t), analysis of the softening degree of the material was used in accordance with Equation (9). Table 5 shows examples of the calculated softening degree of the examined steel for temperatures of 950 and 1050 °C, strain 0.1 and strain rate 10 s^−1^.

**Figure 14:**
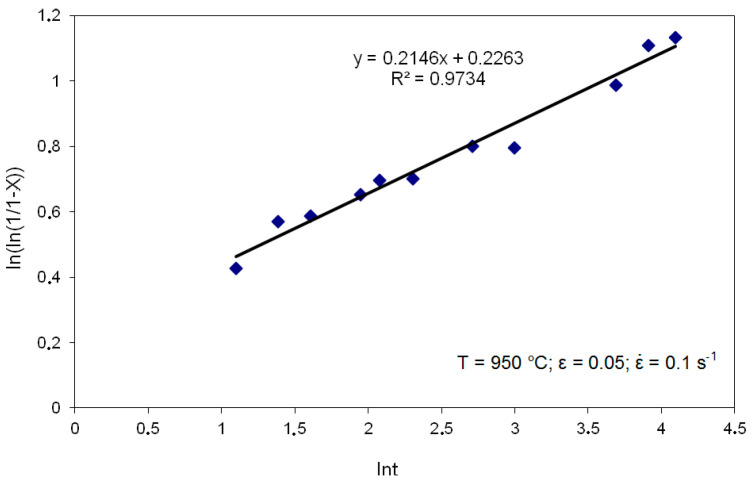
Dependence of ln (ln (1/1 − X)) on ln t for T = 950 °C; ε = 0.05; $\dot{\varepsilon }$ = 0.1s^−1^.

To develop a model of static recrystallization kinetics for the investigated steel, the Avrami equation was used to describe the dependence of the softening fraction as a function of time for individual parameters of the deformation Equation (10). Figure 14 shows the relationship based on which the material coefficients were determined.

For all the deformation values, strain rates and temperatures analyzed at this stage, the values of the coefficients of Equation (10) were determined in accordance with the methodology shown in Figure 14. Table 6 presents exemplary equations with the determined coefficients that describe the change in the proportion of the statically recrystallized phase X in the material with the passage of time (t).

**Figure 15:**
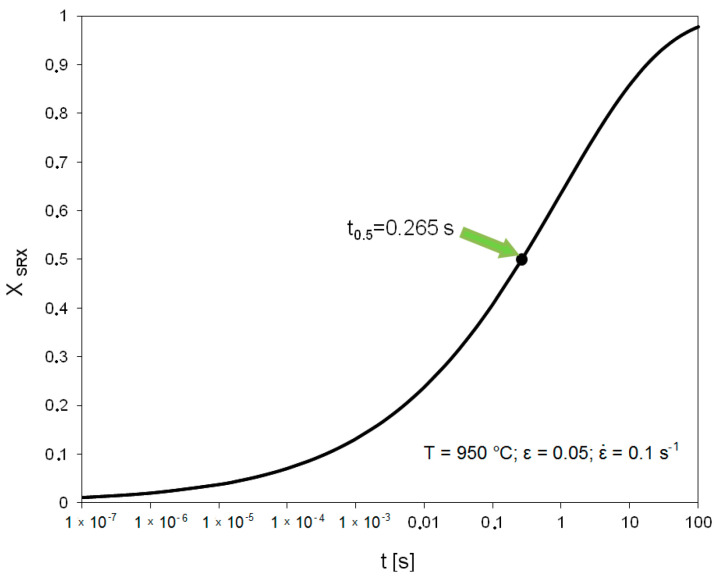
Kinetics of static recrystallization in high-carbon bainitic steel based on Avrami equation.

Figure 15 shows the course of static recrystallization based on the Avrami equation for the temperature of 950 °C, strain 0.05 and strain rate 0.1 s^−1^.

The description of the relationship between the amount of statically recrystallized phase and time by means of equations made it possible to precisely determine the half times of static recrystallization for the given parameters of plastic deformation.

**Figure 16:**
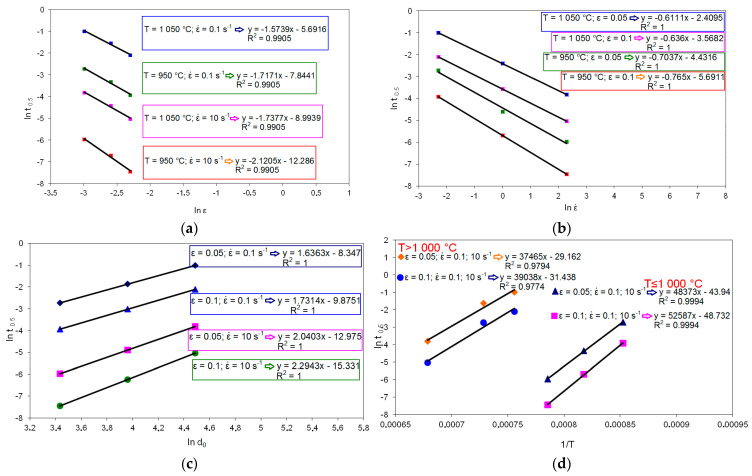
Dependence of half time of static recrystallization (ln t_0.5_) on: (**a**) ln ε, (**b**) ln $\dot{\varepsilon }$, (**c**) ln d_0_, (**d**) 1/T.

In the second step, the coefficients were determined in the model describing t_0.5SRX_ as a function of the strain, strain rate, temperature, and initial grain size. Figure 16 shows the dependencies used to determine the coefficients in the model describing t_0.5SRX_ in high-carbon bainite steel.

The time needed to obtain 50% of the static recrystallized phase in high-carbon bainite steel is described as follows:

for T ≤ 1000 °C

(14) $$t_{0.5_{\mathrm{SRX}}}=6.2828\times 10^{-25}\varepsilon^{-1.9188}\overset{}{{\dot{\varepsilon }}^{-0.7344}d_{0}^{1.9256}e^{\left(\frac{419690.7}{\mathrm{RT}}\right)}}$$

for T > 1000 °C

(15) $$t_{0.5_{\mathrm{SRX}}}=3.0514\times 10^{-21}\varepsilon^{-1.6558}\overset{}{{\dot{\varepsilon }}^{-0.62355}d_{0}^{1.9256}e^{\left(\frac{318023}{\mathrm{RT}}\right)}}$$

**Figure 17:**
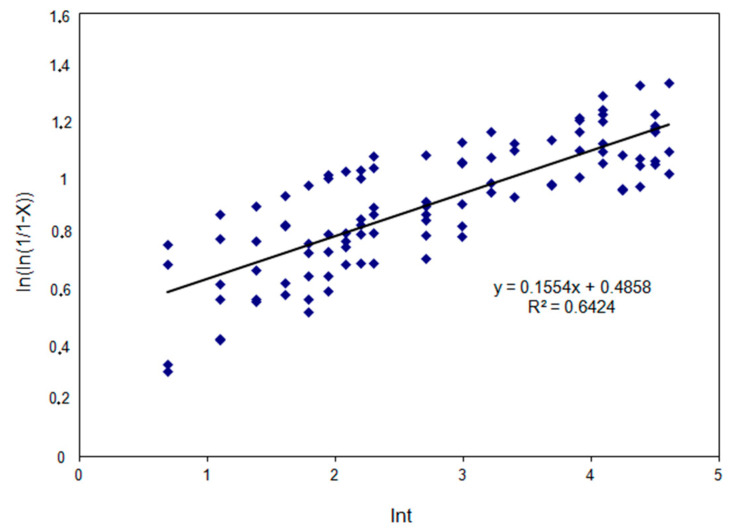
Dependence of ln (ln1/1 − X)) on time (ln t) in range T = 950–1050 °C; ε = 0.05 and 0.1; $\dot{\varepsilon }$ = 0.1 and 10 s^−1^, according to which exponent in model describing kinetics of static recrystallization was determined.

The exponent in the model describing the kinetics of static recrystallization was determined from the relationship presented in Figure 17.

The mathematical model describing the kinetics of static recrystallization in high-carbon bainite steel is as follows:

(16) $$X_{\mathrm{SRX}}=1-\exp\left[-0.6931\cdot {\left(\frac{t}{t_{0.5_{\mathrm{SRX}}}}\right)}^{0.1554}\right]$$

**Figure 18:**
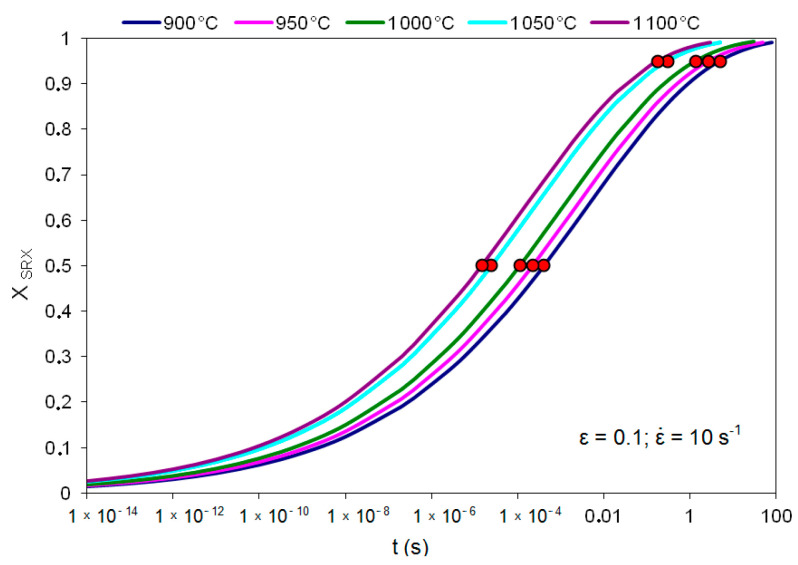
Example of kinetics of static recrystallization in high-carbon bainitic steel based on the developed coefficients in mathematical model.

Figure 18 shows an example of the kinetics of static recrystallization based on the developed model for high-carbon bainitic steel for the temperature range 900–100 °C, strain rate 10 s^−1^ and strain 0.1.

**Figure 19:**
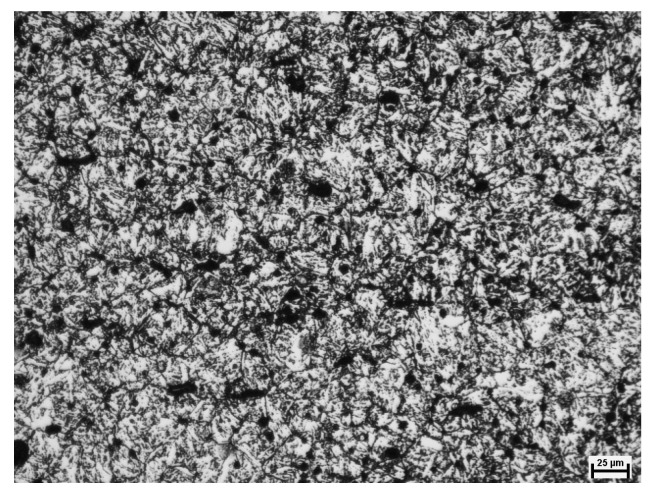
Microstructure of samples after experimental testing for T = 950 °C; ε = 0.1; $\dot{\varepsilon }$ = 10 s^−1^; dγ = 27.2 μm; area 75×; etched with picric acid (C_6_H_3_N_3_O_7_).

To determine the material coefficients of the model describing the grain size of static recrystallized austenite, metallographic tests were carried out after the performed experimental tests. Figure 19 shows an example of the microstructure of static recrystallized alloy austenite grains obtained from the conducted experimental tests.

**Figure 20:**
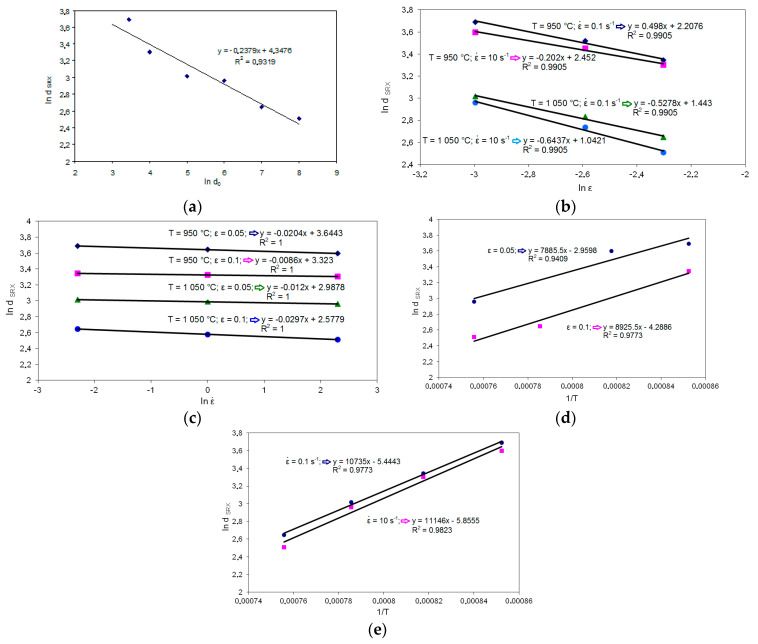
Dependence of statically recrystallized austenite grains (ln _dSRX_) on: (**a**) ln d_0_; (**b**) ln ɛ; (**c**) ln $\dot{\varepsilon }$; (**d**) 1/T for different values of strain, (**e**) 1/T for different values of strain rate.

The mathematical model describing the size of the statically recrystallized grains was developed based on the relationships shown in Figure 20.

Based on the relations presented in Figure 20, the coefficients of the model describing the size of the statically recrystallized grains were determined. The model for high-carbon bainite steel is as follows:

(17) $$d_{\mathrm{SRX}}=7.362\times 10^{-3}d_{0}{}_{}^{-0.2379}\varepsilon^{-0.5224}{\dot{\varepsilon }}^{-0.0177}e^{\left(\frac{80421.32}{\mathrm{RT}}\right)}\;(\mu m)$$

### 3.4. Isothermal Growth of Alloy Austenite Grains

**Figure 21:**
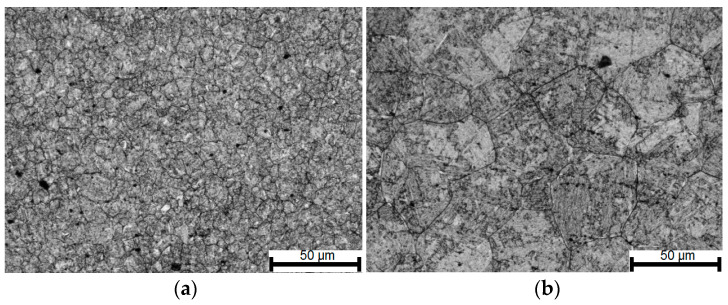
Size of austenite grains after growing at T = 1100 °C: (**a**) after 0 s, d_0_ = 59.7 μm; (**b**) after 3600 s, d_0_ = 98.1 μm; area 100×; etched with picric acid (C_6_H_3_N_3_O_7_).

During the rolling process, deformed and recrystallized austenite grains grow in the gaps between deformations. The research was carried out to develop a model describing grain growth during isothermal annealing. Figure 21 shows sample metallographic photos of samples from the conducted experimental tests.

Based on the measured austenite grain sizes, the growth kinetics are described by the following equation:

(18) $$d{\left(t\right)}^{14.66}=d_{rx}^{14.66}+\left(7.79\times 10^{17}\right)\cdot t\cdot e^{\left(\frac{45279.96}{\mathrm{RT}}\right)}\;(\mu m)$$

### 3.5. The Process of Rolling High-Carbon Bainitic Steel in Industrial Conditions

**Figure 22:**
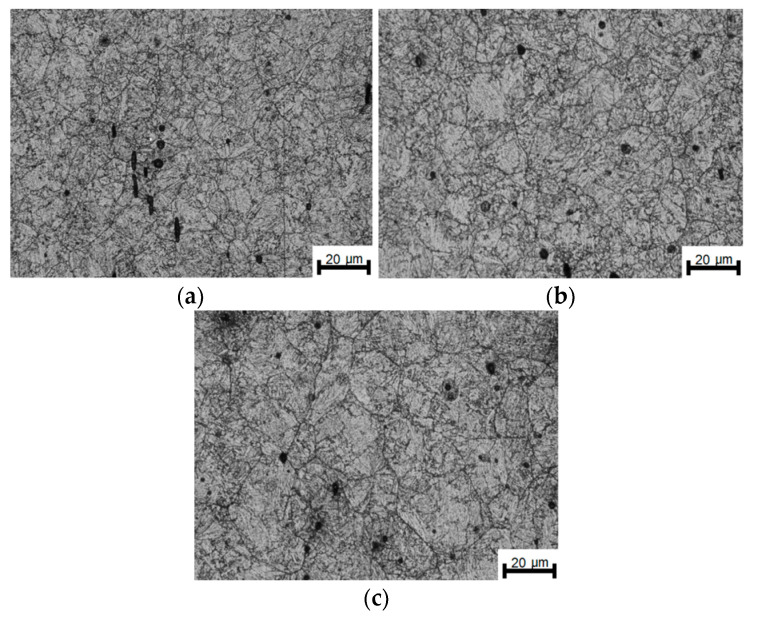
Average grain size: (**a**) variant A: T = 950 °C; dγ. = 26.3 μm, (**b**) variant B: T = 900 °C; dγ. = 28.2 μm; (**c**) variant C: T = 850 °C; dγ. = 29.8 μm. Area 200×; etched with picric acid (C_6_H_3_N_3_O_7_).

The hot-rolling process on a semi-industrial scale was carried out in the LPS rolling line located in the laboratory of the Department of Manufacturing Technology and Application of Products in the Łukasiewicz Research Network—Institute of Iron Metallurgy in Gliwice [10,29]. The rolling process was carried out for three variants: variant A with the finish rolling temperature of 950 °C to obtain full recrystallization in the rolled strip, variant B with the finish rolling temperature of 900 °C in order to obtain partial recrystallization in the rolled strip, variant C with the finish rolling temperature of 850 °C in order to achieve no recrystallization in the rolled strip. Figure 22 presents metallographic photos showing the disclosed size of the alloy austenite grains in the samples from the rolling process according to variants A, B, and C.

**Figure 23:**
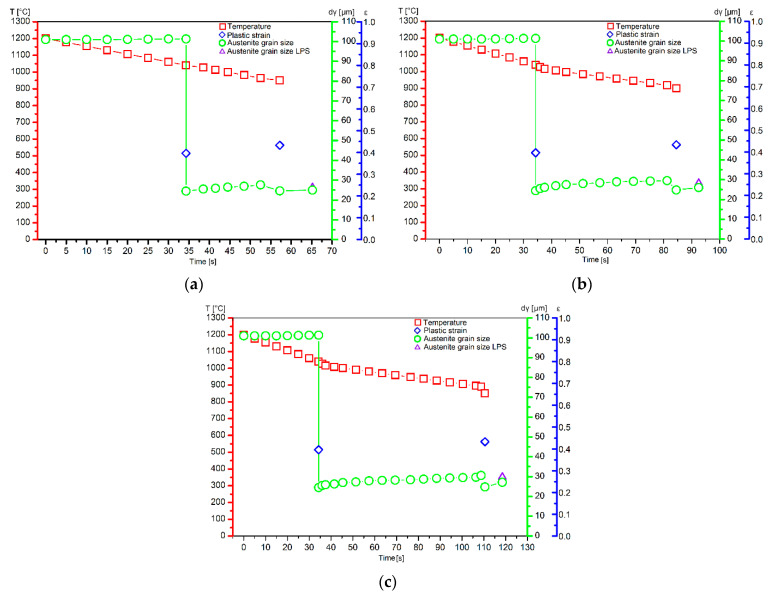
Course of changes in grain size of alloy austenite under conditions of two plastic strains: (**a**) finish rolling temperature of 950 °C, (**b**) finish rolling temperature of 900 °C, (**c**) finish rolling temperature of 850 °C.

Figure 23 displays the course of changes in the average austenite grain size on the time axis during the experimental tests. To analyze the evolution of the alloy austenite, the average values of plastic strain, strain rate, and temperatures were used in the calculations. The course of changes in the austenite grain size was obtained from the implemented models of dynamic, meta-dynamic, and isothermal recrystallization of the grains. Due to the small amount of material intended for actual rolling, it was only possible to compare the amount of recrystallized phase in the steel and the austenite grain size after freezing the structure for 8 s after the completion of rolling. The grain size of the alloy austenite present in the ingot discharged from the rolling furnace was calculated based on earlier research on grain growth and the isothermal grain growth model. It was calculated that after about 50 min. of heating, the grain size is about 102 µm. Moreover, during transport from the furnace to the rolling stand, it undergoes very slight growth, reaching a value of about 104 µm. The deformation set at the temperature of 1040 °C activates dynamic and then meta-dynamic recrystallization of the steel. The effect of these phenomena is a reduction in the average austenite grain size to about 25 µm.

**Figure 24:**
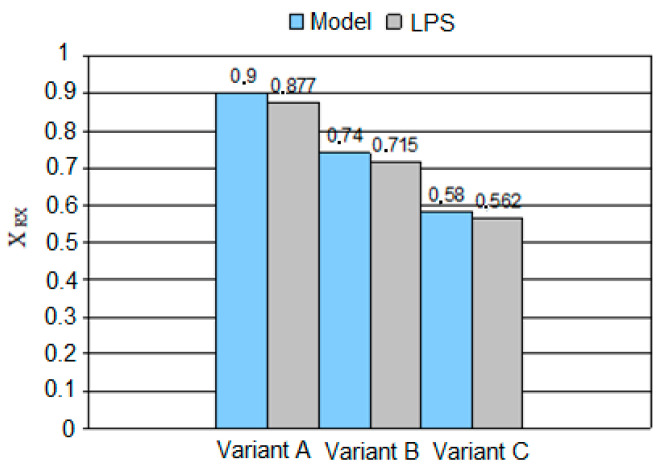
Verification of developed model of microstructure evolution of high-carbon bainitic steel based on experimental tests carried out on semi-industrial LPS rolling line.

In the break between the deformations, which for individual rolling variants is from 18 to 71 s, the grain size increases slightly in the steel. On the other hand, the task of the second deformation causes the activation of recrystallization processes of varying intensity, which in turn leads to obtaining structures of various grain sizes in the steel (Figure 22), while the average austenite grain size in all the studied rolling conditions is similar and amounts to about 28 μm. The grain sizes measured based on the analysis of the metallographic specimens reveal very small differences for the individual variants. It should also be added that considering the difficulties in defining the boundaries of the primary austenite grain and the accuracy of the adopted methods of quantitative analysis of the microstructure, the measurement error should be at the level of about 10%. Comparing the results of measurements of the austenite grain size obtained based on experimental tests and mathematical modeling, it can be concluded that the coefficients appearing in the models describing the size of dynamically and meta-dynamically recrystallized grains were correctly developed. Figure 24 shows the verification of the developed model describing the kinetics of meta-dynamic recrystallization with the results obtained based on experimental tests. Based on quantitative metallography, the percentage of the recrystallized phase of the research samples was determined.

The performed quantitative metallographic analysis of the samples taken from the rolled strands after freezing the structure showed that in the considered variants of the rolling process, different austenite recrystallization occurred. This is the effect of the temperature values adopted in the third pass, at which the plastic deformations were applied. For full verification of the results obtained from numerical models, it would be advisable to conduct more experiments using the LPS line. Unfortunately, it turned out to be impossible due to the lack of rolling material and the high costs of re-melting the rolled sheets and subsequent rolling trials. Nonetheless, for the assumed experimental conditions, the compliance of the amount of recrystallized phase in the steel predicted by the developed numerical models and the final austenite grain size were confirmed.

### 3.6. Study of Mechanical Properties after Heat Treatment

**Figure 25:**
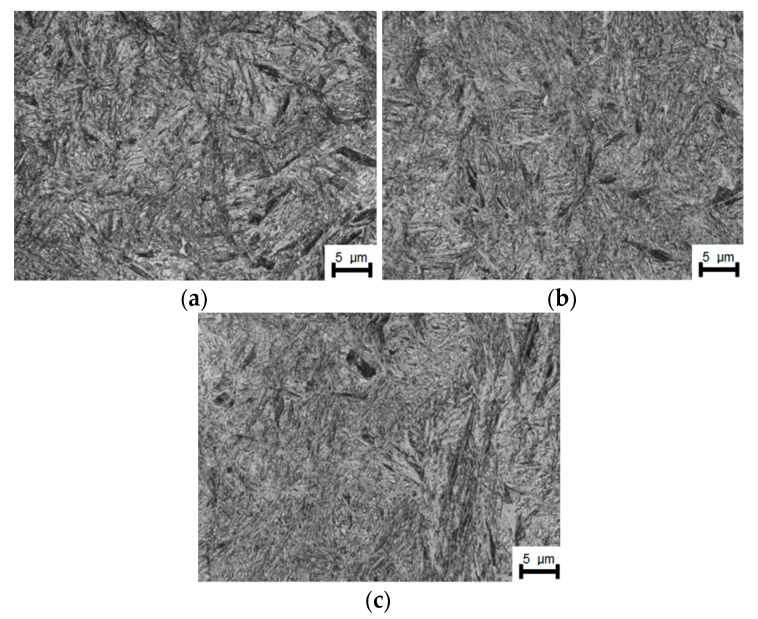
Martensitic-bainitic structure after low-temperature heat treatment at 250 °C for 70 h, area 750×: (**a**) variant A, (**b**) variant B, (**c**) variant C.

Tests of the mechanical properties were carried out after the rolling process to determine whether the refinement of the alloy austenite obtained by recrystallization during hot-rolling has an impact on the mechanical properties of the sheets after heat treatment. Figure 25 presents metallographic photos, based on which the structure of the material was identified after low-temperature heat treatment.

Table 7 and Table 8 show the results of the measurements of Vickers hardness (HV) and tensile strength (TS) after the heat treatment of high-carbon bainitic steel.

The research results presented above indicate that the mechanical properties of the sheets after heat treatment that most depend on the applied rolling scheme, and thus the level of the austenite structure refinement, are the TS and elongation. The maximum TS values were obtained for the sheets at the end of the rolling process at the temperature of 950 °C. The smallest elongation was also noted for these conditions. Nevertheless, taking into account the results of plasticity tests, it can be concluded that 900 °C is the most favorable end temperature of rolling among the one studied. For the sheet rolled in this way, the most favorable plastic parameters were obtained.

## 4. Conclusions

The following conclusions were drawn from the conducted research.

Based on the results of the conducted experimental studies, the coefficients and material constants appearing in the constitutive equations describing the phenomena occurring during hot plastic deformation were developed. Thus, a mathematical description of the kinetics of the dynamic, meta-dynamic, static, and isothermal growth of austenite grains was made. The coefficients and material constants appearing in the equations describing the size of the dynamically, meta-dynamically, and statically recrystallized grains were developed.

The investigated steel undergoes dynamic recrystallization quite quickly with the increase in plastic strain. The value of the critical deformation ε_kr_ at the deformation speed of 0.1 s^−1^ ranges from 0.11 to 0.20 at temperatures from 1100 to 900 °C.

In the examined steel, it was observed that at the temperatures of 950, 1000, 1050, and 1100 °C in the deformation rate range of 0.1÷10 s^−1^, full meta-dynamic recrystallization takes place after times shorter than 10 s. However, for temperatures above 1000 °C, the time after which the full volume of the material recrystallizes can only be estimated based on the model forecast.

Static recrystallization processes are slower than meta-dynamic recrystallization. The times of half-static recrystallization after deformation in the range of 0.05–0.10 at the rate of deformation of 1 s^−1^ are: 1.38 × 10^−4^–0.63 s for temperatures in the range of 1100–900 °C. Due to the relatively low values of critical deformations initiating dynamic recrystallization, the share of static recrystallization in actual plastic working processes is relatively small.

The test rolling carried out in the LPS line provided a relatively small amount of data that could be used to verify the developed models, which was associated with a very limited amount of material for rolling. Notwithstanding, the analysis of the obtained results, especially in the field of quantitative metallography, confirmed the correctness of the forecasting of the developed mathematical models.

The presented results of mechanical tests of sheets after heat treatment show that the TS is dependent on the applied rolling scheme, and thus the level of the austenite structure refinement. The maximum value of TS = 1892 MPa was obtained for variant A with the finish rolling temperature of 950 °C with full recrystallization in the rolled plate. The influence on the final mechanical properties depends on the long-term low-temperature heat treatment resulting in the formation of carbide-free bainite. It is a very long process, and 70 hours of annealing may be not enough to reach full mechanical parameters. Probably therefore we did not obtain the assumed level of TS up to the 2.5 GPa.

## Figures and Tables

**Table 1:** Chemical composition of high-carbon bainitic steel used in tests, mass%.

C	Mn	Si	P	S	Cr	Ni	Mo
0.83	2.20	1.65	0.009	0.014	0.016	0.02	0.39
Co	V	Ti	Al max.	Al. metal.	Cu	N	O
1.58	0.092	0.002	0.039	0.038	0.018	0.0040	0.0009

**Table 2:** Preliminary austenite grain size.

Temperature (°C)	Grain Size (μm)
900	29.1
950	31.7
1000	32.1
1050	89.4
1100	92.8

**Table 3:** Values of softening coefficient under conditions of meta-dynamic recrystallization.

T (°C)	ε (-)	$\dot{\varepsilon }$ (s^−1^)	t (s)	X_m_MDRX__
950	0.28	0.1	1	0.680
			5	0.839
			10	0.916
1000	0.28	0.1	1	0.686
			5	0.951
			10	0.986
1100	0.28	0.1	1	0.894
			5	0.974
			10	0.995

**Table 4:** Kinetics of meta-dynamic recrystallization based on Avrami equation.

T (°C)	ε (-)	$\dot{\varepsilon }$ (s^−1^)	Avrami Equation
900	0.28	0.1	X = 1 − exp(−0.2566t^0^^.1753^)
1000	0.28	0.1	X = 1 – exp(−0.6850t^0.803^)
1100	0.28	0.1	X = 1 – exp(−1.6706t^0.4532^)

**Table 5:** Obtained results of static recrystallization using the stress relaxation method.

T (°C)	ε (-)	$\dot{\varepsilon }$ (s^−1^)	t (s)	$X_{m_{MDRX}}$
950	0.1	10	1	0.884
			10	0.948
			50	0.951
			100	0.979
1050	0.1	10	1	0.912
			10	0.966
			50	0.979
			100	0.994

**Table 6:** Kinetics of static recrystallization based on Avrami equation.

T (°C)	ε (-)	$\dot{\varepsilon }$ (s^−1^)	Avrami Equation
950	0.1	10	X = 1 − exp(−2.0456t^0.1451^)
1050	0.1	10	X = 1 − exp(−2.4133t^0.2138^)

**Table 7:** Average values of Vickers hardness.

Variant	Average Measurement Results	Standard Deviation
A	681 HV10	27.6
B	680 HV10	17.2
C	706 HV10	16.8

**Table 8:** The mean values of the tensile strength.

Variant	Average Measurement Results	
	TS (MPa)	A_5_ (%)
A	1892	1.7
B	1752	2.6
C	1668	2.2

## Data Availability

Data sharing is not applicable to this article.
